# 3D-QSAR and Cell Wall Permeability of Antitubercular Nitroimidazoles against *Mycobacterium tuberculosis*

**DOI:** 10.3390/molecules181113870

**Published:** 2013-11-08

**Authors:** Sang-Ho Lee, Minsung Choi, Pilho Kim, Pyung Keun Myung

**Affiliations:** 1 Drug Discovery Division, Korea Research Institute of Chemical Technology, 141 Gajeongro, Yuseong, Daejeon 305-600, Korea; E-Mails: sangho@krict.re.kr (S.-H.L.); pkim@krict.re.kr (P.K.); 2 College of Pharmacy, Chungnam National University, Daejeon 305-764, Korea; E-Mail: s_ckssar@cnu.ac.kr; 3 Department of Medicinal and Pharmaceutical Chemistry, University of Science and Technology, Daejeon 306-350, Korea

**Keywords:** antitubercular activity, monocyclic nitroimidazoles, *Mycobacterium tuberculosis*, 3D-QSAR, cell wall permeability

## Abstract

Inhibitory activities of monocyclic nitroimidazoles against *Mycobacterium tuberculosis* (Mtb) deazaflavin-dependent nitroreductase (DDN) were modeled by using docking, pharmacophore alignment and comparative molecular similarity indices analysis (CoMSIA) methods. A statistically significant model obtained from CoMSIA was established based on a training set using pharmacophore-based molecular alignment. The leave-one out cross-validation correlation coefficients *q*^2^ (CoMSIA) were 0.681. The CoMSIA model had a good correlation (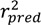/CoMSIA = 0.611) between the predicted and experimental activities against excluded test sets. The generated model suggests that electrostatic, hydrophobic and hydrogen bonding interactions all play important roles for interaction between ligands and receptors. The predicted cell wall permeability (*logP_app_*) for substrates with high inhibitory activity against Mtb were investigated. The distribution coefficient (logD) range was 2.41 < logD < 2.89 for the Mtb cell wall membrane permeability. The larger the polar surface area is, the better the permeability is. A larger radius of gyration (*rgry*) and a small fraction of rotatable bonds (*f_rtob_*) of these molecules leads to higher cell wall penetration ability. The information obtained from the *in silico* tools might be useful in the design of more potent compounds that are active against Mtb.

## 1. Introduction

Tuberculosis (TB) is the World’s second most common cause of death by infectious disease [1]. In 2012, 8.6 million people fell ill with TB and 1.3 million died from [2], which makes TB one of the most dangerous diseases for humans.

It has been estimated that one third of the World’s population is infected with *Mycobacterium tuberculosis* (Mtb), and 10% of these people will become active TB patients during their lifetime [3]. Although first-line and second-line drugs are available in clinics, the emergence of resistant strains of Mtb make TB treatment far more difficult. In fact, it is estimated that half a million cases of new multi-drug resistant (MDR) TB occur every year [2].

The first aerobic antitubercular activity of bicyclic nitroimidazoles was reported from a series of 4- and 5-nitroimidazole[2,1-*b*]oxazoles [4]. Nitroimidazole compounds such as PA-824 currently are in clinical phase II trials [5]. Several studies have identified spontaneously generated PA-824 resistant mutants as a means to understand the cellular machinery involved in its activation [6,7,8,9,10]. The bicyclic nitroimidazole drug sensitivity related to the hypothetical respiratory protein deazaflavin-dependent nitroreductase (DDN) was confirmed, and loss of deazaflavin cofactor F420 biosynthesis ability was reported [11,12], thus the F420 series by glucose-6-phosphate dehydrogenase enzyme (FGD1) activities are dependent [13]. It was known form a series of papers that the F420 dependent glucose-6-phosphate dehydrogenase (FGD1) was sensitive for resistance in isolates and lost the ability to biosynthesize the deazaflavin cofactor F420 [8,9,10]. Rv3547 was essential for susceptibility to the compound and a conserved hypothetical protein encoded by it was identified [12]. FGD1 promotes the oxidation of glucose 6-phosphate to phosphogluconolactone that reduces F420 to F420H2 that is the active form of the cofactor that is utilized by a protein encoded by Rv3547, an enzyme henceforth referred to as a deazaflavin dependent nitroreductase (Ddn) whose physiological role is unknown. F420H2 produces the three stable metabolites from the reduction of the imidazole ring at C-3 in the aerobic state [14]. One of them is the reduced NO, subsequent to the release of nitrous acid DES-nitro-reduced metabolites with antibacterial F420H2 that change to NO from NO_2_ [15]. NO has less *M. tuberculosis* activity than NO_2_, therefore it can easily stay alive in the macrophage [8].

In this study, the structure-activity relationship of monocyclic nitroimidazole analogues against Mtb was investigated by using docking and 3D-QSAR methods. CoMFA and CoMSIA models were obtained based on the pharmacophore alignment. We have predicted the correlation of the Mtb cell wall permeability and their activities in order to understand the cell wall permeability and the Mtb potency properties of nitroimidazole.

## 2. Result and Disscussion

### 2.1. Binding Site and Docking Results

**Figure 1:**
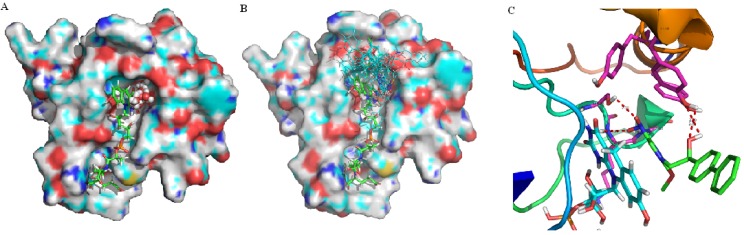
Predicted active site and docked pose for the DDN (PDB 3R5R) of Mtb; (**A**) Predicted active site between DDN and flavine ring of F420, green; F420, sphere; binding site; (**B**) Binding conformations of 21 docked compounds at the active site; (**C**) The interactions between the active binding site and compound **15**.

The solvent-accessible region between DDN and the flavin ring of F420 was predicted by SiteID Pocket Finder. The predicted binding site volume was 11 Å^3^ and the residues were Ser78, Lys79, Gly80, Tyr130, Tyr133 and Tyr136. The docking scores of the binding site inhibitors are listed in Table 1 and represented in Figure 1A. The results of nitroimidazoles docked in the binding site are shown in Figure 1B. A correlation could not be found between docking scores and inhibitory activity (*Obs*.pI_50_). In this result, because the docking scores were often highly correlated with molecular weight, the most significant contributors for scores were nonspecific interactions [16]. Moreover, inhibitory activity for the target was very complicated and affected by some other factors. As a whole the docked forms of the nitroimidazole skeleton into the binding sites were adequately configured and the orientations were similar.

**Table 1:** Observed activities (*Obs.pI_50_*), predicted activities (*Pred.pI_50_*) and docking scores of the Mtb inhibitory activities of nitroimidazoles.

No.	R_1_	R_2_	R_3_	*Obs*.pI_50 _^a^	*Pred*.pI_50 _^b^	Dev. ^c^	DS ^d^
1	2,4-Cl	H		3.98	4.013	−0.033	−7.1
2 ^f^	2,4-Cl	Br		5.28	4.710	0.570	−7.3
3 ^f^	2,4-F	H		3.02	4.164	−1.144	−7.2
4	2,4-F	Br		3.74	3.749	−0.009	−7.6
5	4-F	Br		3.41	3.381	0.029	−7.6
6 ^f^	4-Cl	Br		3.73	3.663	0.067	−7.5
7	4-NO_2_	Br		3.75	3.772	−0.022	−7.1
8	H	Br		3.99	3.950	0.040	−7.2
9 ^f^	2,4-CH_3_	Br		3.42	3.980	−0.560	−7.7
10 ^e^	2,4-Cl	O		5.82	5.886	−0.066	−7.3
11 ^e^	2,4-Cl	O		4.87	4.776	0.094	−7.3
12	4-F	O		4.24	4.266	−0.026	−7.6
13	4-Cl	O		4.27	4.362	−0.092	−7.1
14	4-NO_2_	O		4.29	4.281	0.009	−7.3
15 ^e^	4-Phenyl	O		5.83	5.784	0.046	−7.2
16 ^f^	2,4-Cl	S		4.34	5.380	−1.040	−6.9
17 ^e^	H	O		4.52	4.447	0.073	−7.4
18	2,4-CH_3_	O		4.39	4.429	−0.039	−7.2
19 ^e^	2,4-F		4-Cl	4.42	4.403	0.017	−7.0
20	2,4-F		4-F	4.10	4.121	−0.021	−8.2
21 ^f,g^				6.44	6.020	0.420	−7.6

^a^ Observed inhibitory activity value; ^b^ Predicted inhibitory activity value; ^c^ difference between the observed inhibitory activity and the predicted inhibitory activity; ^d^ Docking score(kcal/mol); ^e^ Template; ^f^ Test set; ^g^ PA-824.

To further explain the interaction among inhibitors, DDN and flavin ring of F420, the most active monocyclic nitroimidazole compound **15** was selected to perform the docking analysis and its ternary complex binding model is displayed in Figure 1C. Hydrogen bond interactions were observed between the O atoms of the NO_2_ group with the NH (2.74 Å) backbone of Ser78 and the OH (1.88 Å) the side chain of Lys79, and between the OH (2.55 Å, 2.69 Å) group of 15 and the OH group of Tyr130.

### 2.2. Pharmacophore and Alignments

**Figure 2:**
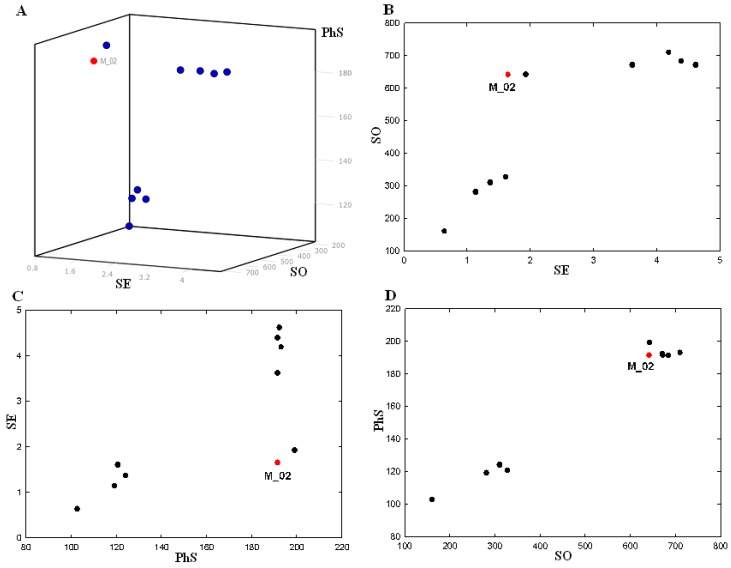
Plot of the strain energy (SE), steric overlap (SO) and pharmacophoric similarity (PhS) values for GALAHAD models with all five ligands with contribution to the consensus feature; (**A**) 3D plot; (**B**) plot of SO *vs.* SE; (**C**) plot of SE *vs.* PhS; (**D**) plot of PhS *vs.* SO.

**Figure 3:**
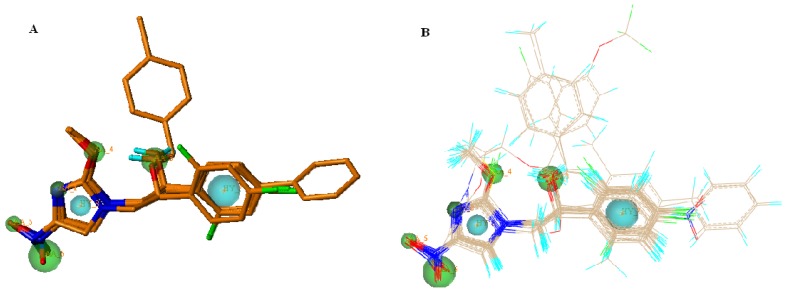
Selected pharmacophore model M_02 (**A**) and molecular alignment (**B**) of the nitroimidazole compounds used to elaborate the model: Cyan, hydrophobes; Green, HB acceptors; Magenta, HB donors.

Pharmacophore models were generated by using the five active ligands in the training set in a GALAHAD run. Twenty pharmacophore models were obtained. Among the twenty generated models, there were 17 Pareto rank 0 models and three 3 Pareto rank 1 models. The Pareto rank 0 is given priority over Pareto rank 1, therefore the Pareto rank 1 models 18, 19 and 20 were excluded from this analysis. All of the 17 models were Pareto rank 0, which means no one model is superior to any other, so we selected one model that met the demands to maximize SO consensus, maximize PhS consensus and minimize low SE. Only the pharmacophore models derived from all five ligands of the training set were compared according to Pareto ranking. Table 2 presents SE, SO and PhS values for models with all five of the ligands. Minimum and maximum values for each characteristic between all the seventy obtained models are also represented in this table. Model 10 had much higher energy than the other nine models, so it is not included in the statistics. A small value of SE, high values of SO and PhS are desired for the best model. SE of the models containing all five ligands (substrates) had a narrow variation value between the minimum (0.33) and the maximum (4.62). SO values varied between the minimum (160.70) and the maximum (710.43), and PhS had a small variation between the minimum (89.40) and the maximum (199.10). With the intention of selecting the optimal model, we represented a 3D and 2D scatter plot to visualize the Pareto Rank in Figure 2. To select the optimal model, a 3D scatter plot was built for the Pareto rank 0 models in Figure 2A. Considering only the SE and SO criteria, the best model lies in the upper left hand corner of the graph in Figure 3B, where the SE is low and the SO is high. In terms of PhS and SE criteria, the best model lies in the lower right hand corner of the graph in Figure 2C, where the SE is low and PhS is high. In terms of PhS and SO, some of the best models lie at the upper right corner, where E as well as PhS are high in Figure 2D. According to Figure 2, there is only one model (M_02), which fulfilled all three requirements described above and it was selected for the subsequent study. This model is represented in Figure 3A. The M_02 model has low SE, and higher SO with high PhS values. Two hydrophobic moieties of the pharmacophore for the monocyclic nitroimidazole analogues were reflected in the presence of hydrophobic structure from the skeleton.

**Table 2:** Strain energy (SE), steric overlap (SO) and pharmacophoric similarity (PhS) values for GALAHAD models with all five ligands with contribution to the consensus feature.

Model	FEATS	SE	SO	PhS
M_01	9	1.93	642.6	199.1
M_02	8	1.65	641.6	191.5
M_03	8	3.62	671.7	191.5
M_04	9	4.39	684	191.5
M_05	8	4.19	710.3	193.1
M_06	8	4.62	671	192.3
M_09	8	1.37	310.4	124.1
M_10	8	1.61	327.4	120.7
M_13	7	1.14	280.9	119.2
M_17	7	0.64	160.7	102.7
Min ^a^		0.64	160.7	102.7
Max ^b^		4.62	710.3	199.1

^a^ Minimum and ^b^ maximum values between all the obtained 17 models.

The HB acceptor moieties represent the importance of these positions in the monocyclic nitroimidazole scaffold for MTB inhibitory activity. Cyan, green and magenta spheres indicate hydrophobes (HY_7, HY_8), HB acceptors (AA_2, AA_3, AA_4, AA_5 and AA_6) and HB donor (DA_1), respectively. It would be possible for us to reduce the number of these pharmacophoric points if we wished to retrieve chemical compounds more distant from the monocyclic nitroimidazole analogues. All conformers aligned represent low-energy conformations of the molecules, and it can be seen that the final alignment shows a satisfactory superimposition of the pharmacophoric points.

All compound datasets were aligned for template M_02 model using the “Align Molecules to Template Individually” option and the other parameters for calculation were set to default values on the GALAHAD run in Figure 3B.

### 2.3. 3D-QSAR

The 3D-QSAR models from CoMFA and CoMSIA analyses were generated and their statistical values are presented in Table 3. For reliable predictive models the cross-validated coefficient q^2^ > 0.5, external predictive (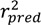) > 0.6 was verified by statistical criteria [17].

The CoMFA model had a cross-validated q^2^ (0.521), a high non cross-validated 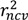 (0.999) and F (1049.253) as well as small SEE (0.032), but it had a high deviation S_cv_ (0.629) and a low external prediction 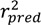 (0.446) which indicates that the CoMFA model was a non-reliable. Therefore we did not consider the CoMFA model.

CoMSIA model analyses using different combinations of Steric (S), Electrostatic (E), Hydrophobic (H), Hydrogen bond Donor (HD), and Acceptor (HA) fields are represented in Table 3. A more statistically meaningful model was obtained from the CoMSIA analyses and these six CoMSIA models all show good correlative and predictive ability. Among the six models, model ID (q^2^ = 0.749,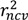 = 0.995) was better than other models for internal CoMSIA statistical values, but the external test set predictive value indicated that the model IC 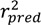 (0.611) was better than ID 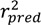 (0.554), in the above criteria with CoMSIA analyses thus the best model IC was selected. The best CoMSIA model included S∙E∙H∙HA fields and had a q^2^ (0.681), 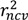 (0.995), F (243.308) and a small SEE (0.067) using six components.

**Table 3:** Summary of statistical results for COMFA and COMSIA models.

Parameters	COMFA	COMSIA					
		IA	IB	IC	ID	IE	IF
Component	6	6	6	6	6	6	6
q^2^^a^	0.521	0.694	0.736	0.681	0.749	0.714	0.671
S_cv_^b^	0.629	0.503	0.467	0.514	0.455	0.487	0.522
r_cv _^c^	0.488	0.655	0.702	0.687	0.722	0.707	0.758
r_ncv_^2 d^	0.999	0.992	0.992	0.995	0.995	0.992	0.994
F ^e^	1049.253	174.433	163.561	243.308	291.917	174.561	214.532
SEE ^f^	0.032	0.079	0.082	0.067	0.061	0.079	0.071
Fraction							
Steric	0.466	0.084	0.095	0.087	0.119		0.096
Electrostatic	0.534	0.425	0.503	0.553	0.708	0.484	0.488
Hydrophobic		0.130	0.147	0.152	0.173	0.143	
Donor		0.199	0.255			0.193	0.233
Acceptor		0.157		0.209		0.180	0.192
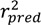 ^g^	0.446	0.516	0.435	0.611	0.554	0.548	0.477

^a^ Leave-one-out cross validated correlation coefficient; ^b^ Leave-one-out cross-validated standard error; ^c^ Conventional correlation of group cross-validation; ^d^ Non-cross-validated correlation coefficient; ^e^ *F*-test value; ^f^ Standard error of estimate of non-cross-validated correlation coefficient; ^g^ External predictivity.

**Figure 4:**
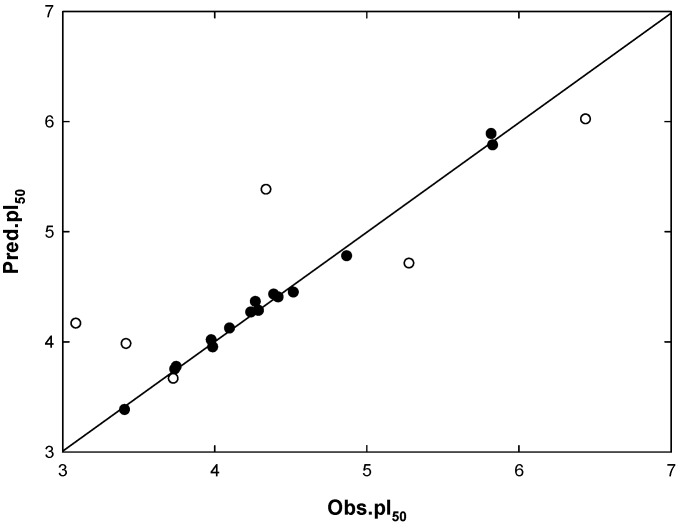
Plot of observed activities (*Obs*.pI_50_) *versus* predicted activities (*Pred*.pI_50_) for the COMSIA model IC; (●) training set; (○) test set.

The CoMSIA model IC indicated a contributions percentage (%) of S (8.7), E (55.3), H (15.1), and HA (20.9), respectively. The E field of the substrate molecule had the highest contribution values of Mtb inhibitory activity. The correlations between the predicted activity values and experimental values are plotted in Figure 4.

**Figure 5:**
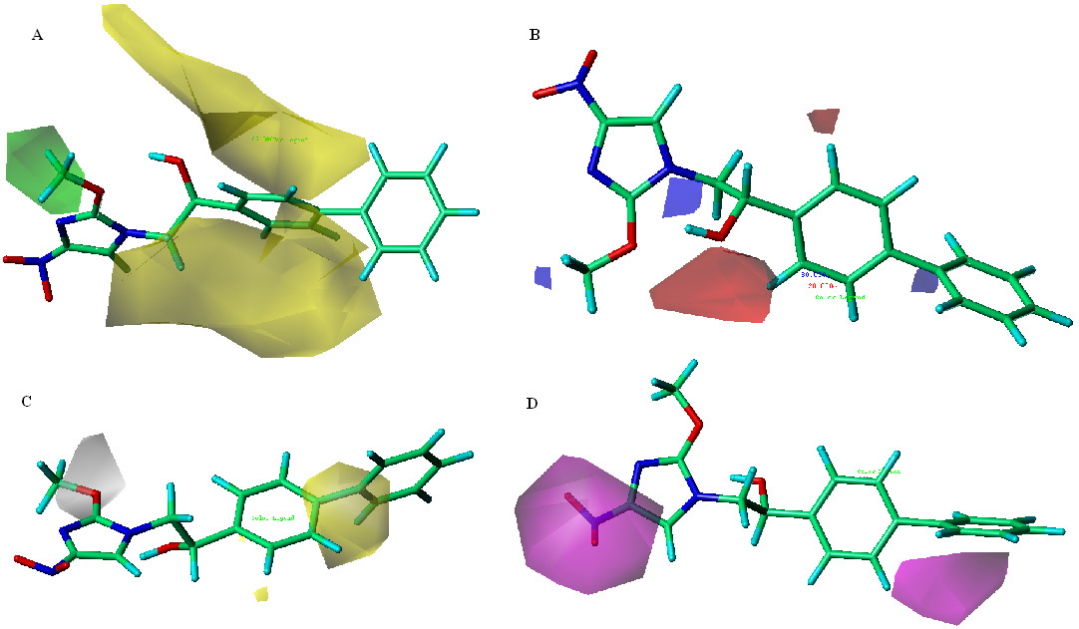
Contour plots with the combination of compound 15 for CoMSIA model IC; (**A**) Steric contour map. Green polyhedrons indicate regions where bulky groups increase activity; Yellow polyhedrons indicate regions where bulky groups decrease activity; (**B**) Electrostatic contour map. Red polyhedrons indicate regions where negative charges increase activity; Blue polyhedrons indicate regions where positive charges increase activity; (**C**) Hydrophobic contour map. Yellow polyhedrons indicate regions where hydrophobic substituent enhances activity; Gray polyhedrons indicate regions where hydrophobic groups decrease activity; (**D**) H-bond acceptor contour map. Magenta polyhedrons indicate regions where H-bond acceptor substituent increases activity.

CoMSIA model IC contour maps are represented in Figure 5, using compound **15** as a reference (template) structure. The Steric field contour map in Figure 5A, is represented by green and yellow polyhedrons in which green polyhedrons indicate regions where a bulky group would be favorable, while the yellow polyhedrons represent regions where a bulky group would decrease the activity. The large yellow polyhedrons (contribution level 15%) are located around ring-A, but the C4-position is separated, indicating that a bulky group in the C2-, C3-positions has decreased inhibitory activity. A small green polyhedron (contribution level 75%) is located in the terminal of the R2 position, indicating that a small steric group has increased inhibitory activity.

The electrostatic field contour map in Figure 5B is indicated by blue and red polyhedrons, which demonstrate the regions where an electron-donating group and an electron-withdrawing group would be favorable. A small blue polyhedron (contribution level 80%) is located in the R2-position and C4-position of ring-A, indicating that the electron-donating group has increased inhibitory activity. Also, the red polyhedrons (contribution level 20%) near the C2-positions of ring-A and OH group location, indicate that the electron-withdrawing group has increased inhibitory activity. In the hydrophobic contour map in Figure 5C, the yellow polyhedron (contribution level 80%) appear near the front of the substituent linking to the C4-position of ring-A in reference compound 15. Accordingly if a positively charged atom is substituented between the ring-A and phenyl group, it improves inhibitory activity because electropositive groups usually exhibit hydrophobic properties. A gray polyhedron (contribution level 20%) enclosing the -O- position of R2 indicates that hydrophilic groups would be favored as a substituent for their activity level because electropositive groups usually exhibit hydrophilic properties. In the hydrogen bond acceptor contour map in Figure 5D, there are two magenta polyhedrons close to the –NO_2_ substituent and the C3-position of ring-A, which indicates that hydrogen bond acceptor groups will increase activity.

### 2.4. Mtb Cell Wall Permeability Prediction

Organic molecules have different ionizable states according to different pH values. When molecules are absorbed into cell membranes, molecule hydrophoicity determines the process rate for membrane permeation. Therefore molecule hydrophoicity is widely used as a predictor of membrane permeability [18]. The influence of ionization on hydrophobicity for a molecule means that the distribution coefficient (logD) should be considered rather than the partition coefficient (logP). The permeation of drugs through the membrane can occur via hydrogen bond cleavage in an aqueous environment. Potentially, molecules can make more hydrogen bonds and it takes more energy to perform these hydrogen bond cleavages, so a high hydrogen bonding potential is an unfavorable property that is often related to low permeability and absorption. Generally, oxygen and nitrogen atoms are molecules that possess high charge density and are electronegative. If the charge density of these atoms is very low, it may have weak hydrogen bonds and electrostatic interactions with other polar atoms. Therefore polar surface area (PSA) is a very significant descriptor for drug transport properties such as membrane permeation and barrier penetration [19]. Molecular weight (MW) and volume are important factors that influence diffusion in biological membranes and continuous fluid media [20]. But the volume and the area of molecular shape are more suitable to describe than MW.

The correlation coefficient between reference HCPSA and calculated PSA are represented in Table 4. It did not list the reference data set descriptors. In the case of model IIB and IIC, an individual correlation coefficient had better PSA (model IIB, r^2^ = 0.497, r = 0.704) than HCPSA (model IIC, r^2^ = 0.463, r = 0.680), and in the case of model IID and IIE, the model IIE had better HCPSA (model IID, r^2^ = 0.624, r = 0.789) than PSA (IIE, r^2^ = 0.609, r = 0.780) in association with logD. However, a correlation coefficient with a very small deviation showed between the multi regression equation of PSA (model IIF, r^2^ = 0.645, r = 0.803) and HCPSA (model IIG, r^2^ = 0.648, r = 0.804) in combination with logD, *rgry* and *f_rtob_*.

Calculated cell wall permeability (*logP_eff_*) and their descriptors of nitroimidazoles are presented in Table 5. The predicted *logP_eff_* of high inhibitory activity compounds (056, 034, 029, and 003) for the MTB was −4.59 < *logP_eff_* < −4.54. From the equation of model IIG, descriptors affecting of logP_eff_ were as follows: *f_rtob_* > logD > PSA > rgry. The *f_rtob_* value range was 0.2222 < *f_rtob_* < 0.2272 and the logD value range was 2.41 < logD < 2.67 against the membrane *logP_eff_* of the high inhibitory activity compounds (056, 034, 029, and 003). However, the range of PSA (127 < PSA < 152) and *rgyr* (3.9282 < *rgry* < 4.8307) showed a large variation.

**Table 4:** Prediction regression model of reference data set.

Parameters	IIA	IIB	IIC	IID	IIE	IIF	IIG
n ^a^	77	77	77	77	77	77	77
q^2 b^	0.475	0.468	0.436	0.594	0.583	0.597	0.598
r^2^^c^	0.497	0.497	0.463	0.624	0.609	0.645	0.648
r	0.704	0.704	0.680	0.789	0.780	0.803	0.804
F ^d^	73.98	74.03	64.68	61.29	57.51	67.20	67.98
SEE ^e^	0.537	0.537	0.554	0.467	0.477	0.454	0.450
logD ^f^	0.313			0.212	0.192	0.225	0.200
PSA ^g^		−0.006			−0.004		−0.004
HCPSA ^h^			−0.011	−0.007		−0.007	
rgyr ^i^						−0.082	−0.130
f_rtob_^j^						0.431	0.362
c ^k^	−5.261	−4.313	−4.278	−4.685	−4.707	−4.497	−4.313

^a^ Number of compounds for reference data set; ^b^ Leave-one-out cross validated correlation coefficient; ^c^ Non-cross-validated correlation coefficient; ^d^ *F*-test value; ^e^ Standard error estimate of non-cross-validated correlation coefficient; ^f^ Distribution coefficient; ^g^ Polar surface area; ^h^ High charged polar surface area; ^i^ Radius of gyration; ^j^ Fraction of rotatable bonds; ^k^ Constant.

**Table 5:** Molecule descriptors and predicted nitroimidazoles *logP_eff_*. for Mtb cell walls.

No.	*logP_eff_* ^a^	*rgyr* ^b^	*f_rtob_* ^c^	logD ^d^	PSA ^e^
1	−4.6116343	4.0313	0.2000	2.46	149.838
2	−4.5404066	3.9282	0.1904	2.67	147.268
3	−4.8524414	3.8254	0.2000	1.39	152.410
4	−4.7692208	3.6884	0.1904	1.59	145.796
5	−4.7926759	3.7089	0.2000	1.50	147.788
6	−4.7023681	3.9511	0.2000	2.05	150.179
7	−5.0909159	4.0777	0.2272	1.24	239.008
8	−4.7872804	3.4399	0.2105	1.31	150.507
9	−4.6492473	3.6979	0.1904	2.15	156.862
10	−4.5570507	4.0205	0.2272	2.41	127.883
11	−4.8030476	3.8244	0.2272	1.33	130.829
12	−4.8236766	3.8606	0.2380	1.24	130.382
13	−4.7282331	4.0861	0.2380	1.79	131.800
14	−5.1202633	4.2235	0.2608	0.98	219.603
15	−4.5974279	4.8307	0.2222	2.89	130.405
16	−4.4095507	4.0205	0.2272	3.09	141.513
17	−4.8078457	3.5847	0.2500	1.05	129.134
18	−4.6659152	3.8600	0.2272	1.89	130.823
19	−4.0633268	4.0268	0.2580	4.05	103.310
20	−4.1658948	4.2228	0.2580	3.79	102.466
21	−4.5433115	4.2727	0.2222	2.86	152.777

^a^ Predicted Mtb cell wall permeability; ^b^ Radius of gyration; ^c^ Fraction of rotatable bonds; ^d^ Distribution coefficient; ^e^ Polar surface area.

Recently, qualitative physochemical properties of preclinical and clinical development anti-TB drugs have been predicted with regard to the cell wall permeability using an *in silico* method [21]. The predicted average parameter values were clogP (3.5), MW (431.4), HA (6), HD (1) and rotatable bonds (RB) (6). The high activity monocyclic nitroimidazole compound **15** also has logD (2.89), MW (339), HA (6), HD (1) and RB (7) values. We thus found that the cell wall permeability prediction average parameter values were very similar to those of the high activity compound **15**.

## 3. Experimental

### 3.1. Data Set

3D QSAR studies were performed on the Mtb inhibition of monocyclic nitroimidazole analogues, where the observed inhibitory activity (*Obs*.pI_50_) values were calculated from previously reported data [22]. The minimum inhibition concentration (MIC) values of some molecules were qualitatively (they do not have exact activity quantities) higher than 256 μM. These molecules were removed from the data set. For the QSAR analysis the MIC values were converted to 50% inhibitory molar concentrations and were expressed in negative logarithmic units. The *Obs*.pI_50_ was calculated from following equation (1):


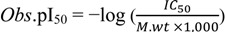
(1)

**Figure 6:**
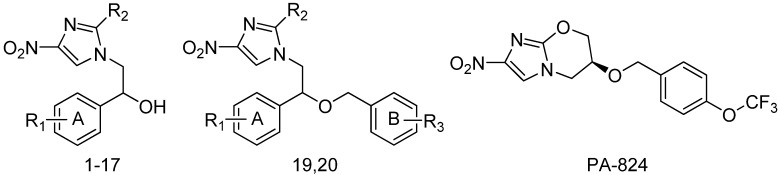
General structures of the monocyclic nitroimidazoles and PA-824(**21**).

The inhibitory activities (*Obs*. pI_50_) against Mtb with substituent changes in the nitroimidazole derivatives **1**–**21** (Figure 6) were determined. The chemical structures and corresponding *Obs*.pI_50_ values are listed in Table 1.

### 3.2. Predicted Binding Sites and Docking Simulation of DDN

The crystal structure of DDN (PDB 3R5R) was taken from the Protein Data Bank (PDB). The unidentified ternary complex of DDN with F420 was found but none with inhibitor [23]. The binding site was predicted from the generated solvent accessible region by the Sybyl module SiteID [24] and the cavity centroid, determined as an appropriate distance from C5 of the flavin ring for hydride transfer [23,25]. AutoDock (Ver. 4.2) [26] was used for the active site of the protein and was defined by a centered grid of 60 × 60 × 60 points with a grid spacing of 0.35 Å. The Lamarckian Genetic Algorithm (LGA) was used with 30 runs, and a clustering of docked poses was applied according to the RMSD. The best pose with the lowest AutoDock score on the top cluster was retained for each compound.

### 3.3. Pharmacophore Model and Molecular Alignment

All structures obtained from docking were imported into SYBYL-X2.0 [24] as mol2 files and assigned Gasteiger-Huckel atomic partial charges. Pharmacophore models 10, 11, 15, 17 and 19 from the template data set compounds were selected by the distribution for biological activities and structural diversity that were generated using a genetic algorithm with a linear assignment for the hypermolecular alignment of datasets (GALAHAD) module. GALAHAD was run for 100 generations with a population size of 70 and a tournament pool size of 250. Default values were used for other settings, and the best model from a low strain energy (SE), steric overlap (SO), and pharmacophoric similarity (PS) in the Pareto ranking of the generated models was selected. All compounds were aligned to the best pharmacophore model using the Align Molecules to Template Individually method. 

### 3.4. 3D-QSAR Models

The CoMFA and CoMSIA models that were generated from the analytical correlation results between descriptors of the structural character of inhibitors and their Mtb inhibitory activities were derived using partial least square (PLS) analysis [27]. The set of inhibitors were divided into a training and test set. Training set compounds (n = 15) were derived from all data set compounds (n = 21) and test set compounds (n = 6), then predictabilities of each model using a test set were discussed.

The CoMFA fields were generated using steric and electrostatic probes with standard 30 kcal/mol cut-offs. In the CoMSIA analyses, similarity was expressed in terms of steric occupancy, electrostatic interactions, local hydrophobicity, and H-bond donor and acceptor properties, using a 0.3 attenuation factor.

The PLS method [28] was used to correlate the CoMFA and CoMSIA fields to *Obs*.pI_50_ of inhibitory activity values. The generated models were assessed using a leave-one-out (LOO) cross-validation procedure by the SAMPLS method as implied in the PLS module. The LOO is a method in which one compound is removed from the dataset and its activity is predicted using the model derived from the rest of the molecules in the dataset. A strict criterion for the selection of the optimal number of components was applied by selecting the lowest PRESS value and the less complex model was chosen with the corresponding cross-validation (CV) coefficient (q^2^) [29]. The q^2^ value resulted in a minimal number of components and the lowest CV standard error (SE, S_cv_) of estimate was accepted. The column filtering values (σ_min_) were set to 2.0 kcal/mol in order to speed up the analytical process and reduce noise. The optimal number of components were used to derive the final PLS model, with a non-cross-validation (ncv) method [30]. The higher correlation (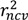)and predictability (q^2^ or 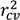) among derived models was selected to test the utility of the model as a predictive tool. The prediction of the model between training sets (internal) and test sets (external) was calculated from according to equation (2):

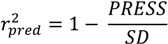
(1)
where PRESS is the sum of the squared deviations between predicted and actual pI_50_ values for the test set compounds. SD is the sum of the squared deviation between the actual pI_50_ values of the compounds from the test set and the mean pI_50_ value of the training set compounds. The best model from a correlation coefficient (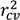) > 0.50, coefficient of determination (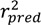) > 0.60 [17] for the two statistical criteria was selected. To analyze the visualized structural distinctions of inhibitors, information from the best model was expressed in three dimensional space on contour maps (steve*coeff).

### 3.5. MTB Cell Wall Permeability Prediction

The Caco-2 cell membrane permeability models were generated from descriptors of the structural characteristics of nitroimidazoles. The distribution coefficient (*logD*, CDLab ver 6.0) [31] of hydrophobicity at pH 7.4, MOLPROP_PSA (PSA) [24] of hydrophilicity, radius of gyration (*rgyr*) (E-Dragon) [32] and fraction of rotatable bonds (*f_rotb_*) (JOELib) [33] of molecular bulkiness were calculated. A training data set of reference data was obtained from the literature [34,35]. The correlation between the reference data high charged polar surface area (HCPSA) and the calculated PSA was compared using Sybyl PLS analysis. The Caco-2 cell membrane permeability value of nitroimidazoles from this data set was calculated and Mtb cell wall permeability (*logP_eff_*) of inhibitors was predicted using this data.

## 4. Conclusions

In this study, the docking, pharmacophore model, 3D-QSAR and cell wall permeability properties were obtained to predict the Mtb inhibition activity of nitroimidazoles, which is important information to support the design of active compounds. The hypothetic binding orientations of compounds interacting with DDN and F420 were revealed by the docking studies, and a pharmacophore model obtained using GALAHAD is a useful molecular alignment tool for measuring Mtb inhibitory activity and was able to produce a good 3D-QSAR model. Pharmacophores consisted of one donor atom, five acceptor atoms, and two hydrophobic centers in a monocyclic nitroimidazole. The CoMSIA model was established using pharmacophore-based molecular alignment and the statistical significance of the model was evaluated. The CoMSIA model was stable and had statistically significant predictive ability. High correlation and predictability were established (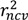 = 0.995, q^2^ = 0.681) and the predictive correlation coefficient (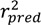 = 0.611) for the test set determined. The Mtb cell wall permeability was predicted through Caco-2 cell permeability. The distribution coefficient ranges were 2.41 < logD < 2.89 for the Mtb cell wall permeability. A combined docking, pharmacophore searching and 3D-QSAR study can thus effectively direct drug molecular design.
